# Bioactivation of nitroglycerin is determined by the subcellular localization of aldehyde dehydrogenase-2

**DOI:** 10.1186/2050-6511-13-S1-A21

**Published:** 2012-09-17

**Authors:** Regina Neubauer, Corina Madreiter, Matteo Beretta, Gerald Wölkart, Kurt Schmidt, Astrid Schrammel, Bernd Mayer

**Affiliations:** 1 Department of Pharmacology and Toxicology, Karl-Franzens University Graz, 8010 Graz, Austria

## Background

Aldehyde dehydrogenase-2 (ALDH2) was characterized as the main enzyme responsible for bioactivation of the antianginal drug nitroglycerin (GTN). We have recently shown that ALDH2 is mainly cytosolic in murine vascular tissue, challenging the general assumption that GTN bioactivation takes place in the mitochondrial matrix of vascular smooth muscle cells.

## Objective

In the present study we investigated whether bioactivation of GTN is affected by the subcellular localization of ALDH2 using immortalized ALDH2-deficient aortic smooth muscle cells with selective overexpression of the enzyme in either cytosol or mitochondria. Furthermore we investigated a potential correlation between the relaxation potency of GTN and the distribution of ALDH2 in arterial blood vessels from different species as well as the subcellular distribution of the enzyme in several murine organs.

## Methods and results

Radio thin layer chromatography analysis showed that cytosolic overexpression of ALDH2 led to denitration rates up to 4 times higher than mitochondrial overexpression, suggesting a more efficient bioactivation by cytosolic ALDH2. Interestingly, denitration rates of smooth muscle cells were even higher in cells without functional mitochondria (Rho0 cells), suggesting possible adverse effects of mitochondria on the bioactivity of GTN. Quantitative immunoblotting revealed that ALDH2 is mainly cytosolic in murine, rat, guinea-pig and rabbit aortas as well as in porcine, bovine and human coronary arteries. A similar expression pattern was found in several murine organs, except liver. Cumulative concentration-response curves to GTN established by vasorelaxation studies were biphasic for aortas with more than 10% ALDH2 in mitochondria (mouse and rabbit), strengthening the hypothesis that mitochondrial GTN metabolism counteracts cytosolic bioactivation of the drug.

## Conclusions

The data indicate that cytosolic expression is essential for GTN bioactivation in arterial blood vessels and aortic smooth muscle cells, presumably due to limited access of GTN to the mitochondrial matrix.

